# Promotion of SH-SY5Y Cell Growth by Gold Nanoparticles Modified with 6-Mercaptopurine and a Neuron-Penetrating Peptide

**DOI:** 10.1186/s11671-017-2417-x

**Published:** 2017-12-29

**Authors:** Yaruo Xiao, Enqi Zhang, Ailing Fu

**Affiliations:** 1 College of Bioengineering, Chongqing University, Chongqing, 400044 People’s Republic of China; 2 College of Pharmaceutical Sciences, Southwest University, Chongqing, 400715 People’s Republic of China

**Keywords:** 6MP-AuNPs-RDP, Cell proliferation, Neurite growth

## Abstract

Much effort has been devoted to the discovery of effective biomaterials for nerve regeneration. Here, we reported a novel application of gold nanoparticles (AuNPs) modified with 6-mercaptopurine (6MP) and a neuron-penetrating peptide (RDP) as a neurophic agent to promote proliferation and neurite growth of human neuroblastoma (SH-SY5Y) cells. When the cells were treated with 6MP-AuNPs-RDP conjugates, they showed higher metabolic activity than the control. Moreover, SH-SY5Y cells were transplanted onto the surface coated with 6MP-AuNPs-RDP to examine the effect of neurite development. It can be concluded that 6MP-AuNPs-RDP attached to the cell surface and then internalized into cells, leading to a significant increase of neurite growth. Even though 6MP-AuNPs-RDP-treated cells were recovered from frozen storage, the cells still maintained constant growth, indicating that the cells have excellent tolerance to 6MP-AuNPs-RDP. The results suggested that the 6MP-AuNPs-RDP had promising potential to be developed as a neurophic nanomaterial for neuronal growth.

## Background

Promotion of neuronal cell proliferation and neurite growth is important in nerve regeneration [1, 2], for which much efforts have been made in order to treat neurodegenerative diseases such as Alzheimer’s disease (AD), Parkinson’s disease (PD), and strokes [3, 4]. It was demonstrated in a number of studies that surface properties of materials could affect cell morphology, even interfere/promote cell replication and differentiation, which hold great promises in regenerative medicine and developing a new strategy of nanomaterials with active biological functionality as neurophic agents [5, 6].

Among the existing biomaterials, gold nanomaterials are used in a wide range of biological applications including sensing, labeling, drug delivery, and imaging, due to their ease of synthesis, convenience for surface functionalization, low toxicity, good stabilization, and biocompatibility [7, 8]. For example, a previous study reported that gold nanorods associated with low-power laser exposure stimulated the increase of neurite length up to 25 μm of NG108-15 neuronal cells compared with the control [9].

**Fig. 1:**
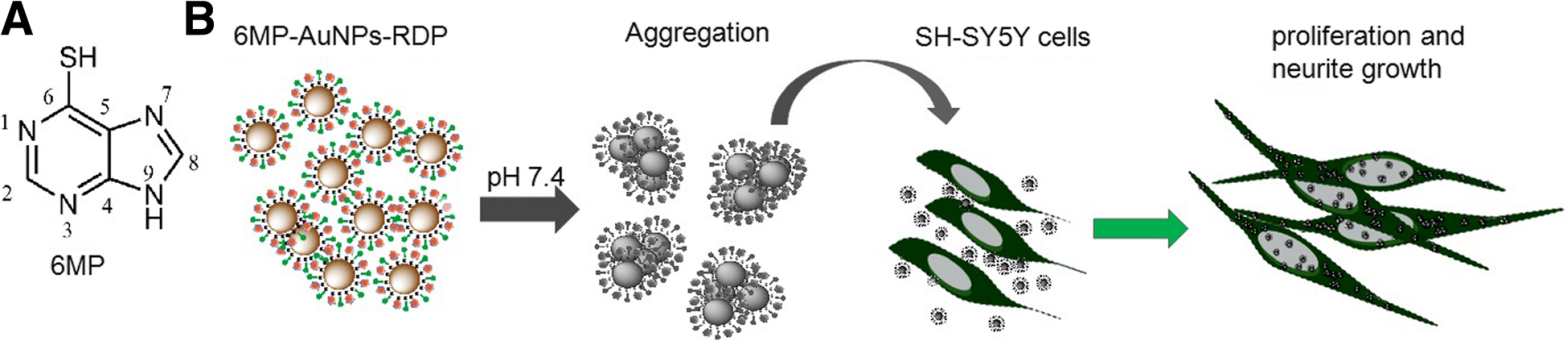
Scheme of experimental procedure. **a** 6-Mercaptopurine structure. **b** Experimental procedure. The particles were synthesized at pH 9.0 and appeared aggregated at pH 7.4. When the particles were added into the SH-SY5Y cell media, they were internalized into the cells and stimulated cell growth

6-Mercaptopurine (6MP; Fig. 1a), an anti-inflammatory drug, has been used to functionalize the surface of gold nanoparticles (AuNPs) to form 6MP-modified AuNPs (6MP-AuNPs) through an Au-sulfur bond [10]. It was reported that 6MP-AuNPs were used to quantitatively analyze 6MP concentration in solvent through a turn on-and-off mechanism [11]. However, no data shows the effect of 6MP-AuNPs on cells.

Here, we used neuronal cell line to investigate the interaction of 6MP-AuNPs and cells, since it is well known that neuronal cells are strongly affected by the property of culture substrates. Among neuronal cell lines, human neuroblastoma (SH-SY5Y) cell is considered as a widely used model system due to its high sensitivity to environmental stimulation and importance for functional biomaterials in neural research. Moreover, in order to increase the neural cell uptake efficiency of 6MP-AuNPs, a neuron-targeting peptide (RDP) was linked to the particle surface to form a 6MP-AuNPs-RDP conjugate. The results suggested that the conjugate showed an obvious neurophic activity, but not anti-proliferative effect of 6MP, leading to a significant increase in cell proliferation and neurite growth.

## Methods/Experimental

### Synthesis of 6MP-AuNPs-RDP Conjugate

Citrate-coated AuNPs with the size of 20 nm were synthesized by reduction method. Briefly, an aqueous solution of HAuCl_4_·3H_2_O (100 mL, 0.01%) was heated with vigorous stirring for 30 min, then citrate sodium solution (10 mL, 38.8 mM) was quickly added into the HAuCl_4_ solution. The mixture was refluxed for another 30 min, until a deep red solution was obtained, and cooled to room temperature naturally.

6MP-AuNPs were prepared by mixing AuNPs (0.33 mM) and 6MP solution (final concentration 0.046 nM) for 5 h at room temperature according to a previous report [12]. Then, the mixture was centrifuged at 17,000*g* for 30 min. Subsequently, supernatant was discarded, and pellet (6MP-AuNPs) was resuspended and washed with deionized water for three times.

To obtain RDP-modified 6MP-AuNPs, RDP (FAM w/ CKSVRTWNEI IPSKGCLRVG GRCHPHVNGG GRRRRRRRRC; synthesized by Shanghai Ji’er Biotech. Co., China) and 6MP, with respective final concentration of 0.023 nM, was simultaneously added to the AuNP solution (0.33 mM) for 5 h and then centrifuged at 17,000*g* for 30 min. As a control, a FAM-labeled scramble peptide (FAM-SP; GRNECRIPRV GCVSRWRIGR KGRCHRLRPG GRVNRSHT GC) was synthesized (Shanghai Ji’er Biotech. Co., China) and 6MP-AuNPs-SP-FAM was prepared in parallel. Afterwards, supernatant of the particles was discarded, and the particles were respectively washed with deionized water. The particle solutions were respectively adjusted to pH 9.0 with 0.1 M NaOH and then passed through 0.22-μm syringe filters and were stored at 4 °C for use.

### Characteristics of the Particles

Absorption spectra were measured at room temperature with a UV/vis spectrophotometer (UV-2450, Shimadzu Corp, Kyoto, Japan) to detect optical absorption of the particles. Particle size and zeta potential of the particles were respectively measured by using a dynamic light-scattering (DLS) apparatus (Zetasizer Nano ZS; Malvern) after dilution with deionized water. Transmission electron microscopy (TEM; Shimadzu) was used to observe the particle structure.

### Cell Culture

SH-SY5Y cells were cultured in Dulbecco’s modified Eagle’s medium (DMEM) and F-12 medium with the ratio of 1:1. The media were respectively supplemented with 10% fetal calf serum (FCS), 100 units/mL penicillin, and 100 μg/mL streptomycin. The cells were maintained at 37 °C with 5% CO_2_ in a humidified incubator (Thermo Fisher Scientific, USA). All reagents for cell culture were purchased from HyClone (USA).

### Cell Uptake

SH-SY5Y cells were seeded into 24-well plates at a density of 5 × 10^4^ cells/well. When cell confluence reached 60%, FAM-labeled 6MP-AuNPs-RDP and 6MP-AuNPs-SP of final concentration 0.25 μg/mL were respectively added to the cell media for a 2-h incubation. Then, the cell media were discarded and replaced with fresh media. The cells were observed and photographed by using a fluorescence microscope (Olympus, Japan).

### Impact of 6MP-AuNPs-RDP on Neuronal Growth

SH-SY5Y cells were seeded into 24-well plates at a density of 5 × 10^5^ cells/well overnight. Then, RDP-6MP-AuNPs with different concentrations (0, 0.125, 0.25, 0.5, 1.0 μg/mL) were respectively added into the media for a 24 h incubation. Cell numbers were counted by using an automated cell counter (Bio-Rad, USA).

Also, cell metabolic activity was measured by 3-(4,5-dimethylthiazol-2-yl)-2,5-diphenyltetrazolium bromide (MTT) assay according to the previous report [13]. Briefly, SH-SY5Y cells were seeded into 96-well plates at a density of 5 × 10^4^ cells/well and incubated in media containing 10% FBS for overnight. Then, the particles were respectively added into the media for 24 h. The cells were washed with PBS for three times, then 100 μL fresh media and 10 μL MTT (5 mg/mL in PBS buffer) were added to each well. Following a 4-h incubation, the media were removed and 200 μL dimethyl sulfoxide (DMSO) was added to dissolve the produced formazan. The absorbance of supernatant was measured at 490 nm using a microplate reader (Bio-Rad, USA). Cells without any additions are used as blank, and the cells with only solvent (0.1 M NaOH (pH 9.0) were adjusted to pH 7.4 by 0.1 M HCl) as the control. The relative cell metabolic activity was calculated as metabolic activity (%) = OD_490_ (sample-blank)/OD_490_ (control-blank). Each value was averaged from four independent experiments.

To determine the effect of 6MP-AuNPs-RDP on neurite growth, SH-SY5Y cells were transplanted into 6-well plates and grown to 30% confluence. Then, the cells were treated with 6MP-AuNPs-RDP (0.25 μg/mL) once a day for 3 days. The neurite lengths were observed under an optical microscopy (Olympus, Japan) and calculated by using an ImageJ software [14].

### Cell Proliferation on the Surface Coated with 6MP-AuNPs-RDP

6MP-AuNPs-RDP were plated homogenously onto the bottoms of culture dishes of 3.5 cm diameter, then the cells were transplanted onto the particle-coated dishes. After incubation, the cells were observed under the optical microscopy and neurite length was counted. The cells with only solvent were used as a control. Each experiment was repeated four independent times, and 200 neurites were averaged for calculation of neurite length.

### Statistical Analysis

Data were expressed as mean ± SEM. The data were analyzed with a computer program by one-way analysis of variance (ANOVA), followed by Dunnett’s multiple range test, with SPSS 13.0 software. Differences with *p* < 0.05 were considered statistically significant.

## Results

### Appearance and Characteristics of the Nanoparticles

**Fig. 2:**
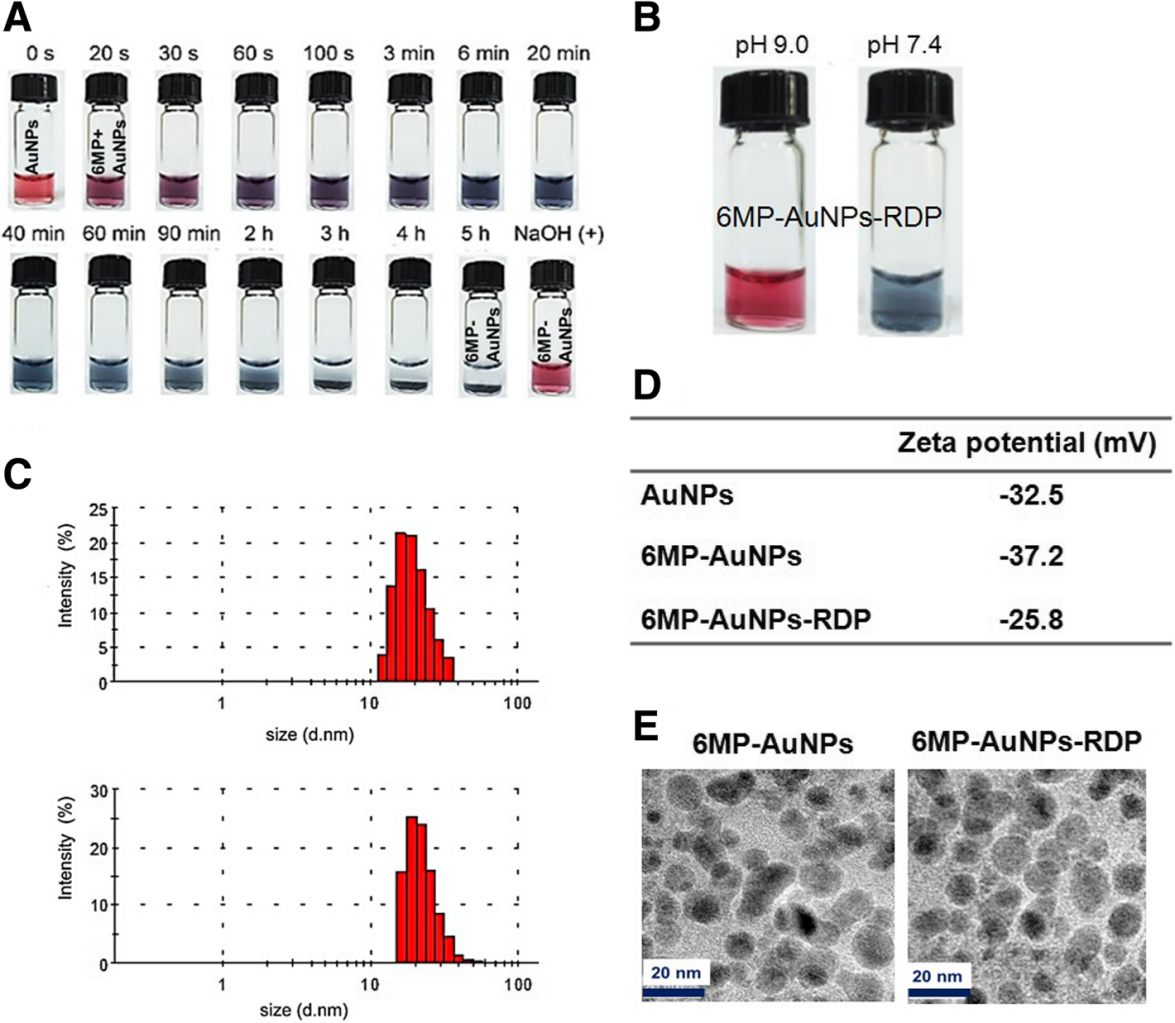
Reaction process and characteristics of the nanoparticles. **a** Reaction process for preparing 6MP-AuNPs. After 6MP was added into AuNP solution, the solution color changed and precipitation was gradually formed in 5 h. **b** 6MP-AuNPs-RDP precipitation was dissolved when pH was adjusted to 9.0 by 0.1 M NaOH. **c** DLS measurement of size distribution of the particles. **d** Zeta potential of AuNPs, 6MP-AuNPs, and 6MP-AuNPs-RDP. **e** Particle structures under TEM

The aqueous solution of AuNPs showed a scarlet color under visible light (Fig. 2a, 0 s). After the addition of 6MP, the color gradually became dark when 6MP was conjugated to AuNPs, and finally, a blue-black precipitation of 6MP-AuNPs appeared after 5 h of reaction. The precipitation could be resolved by adjusting the pH to 9.0, and at the time, the aqueous solution of 6MP-AuNPs showed a rose color. Precipitation would be re-formed when pH was adjusted to pH 7.4.

6MP-AuNPs-RDP was prepared by conjugating the AuNPs with thiol groups of 6MP or RDP at pH 9.0. The aqueous solution of 6MP-AuNPs-RDP showed the same rose color as that of the 6MP-AuNP solution (Fig. 2b). When the pH of 6MP-AuNPs-RDP solution was adjusted to 7.4, the particles precipitated at the bottom of the solution.

Size and zeta potential of 6MP-AuNPs and 6MP-AuNPs-RDP solution (pH 9.0) were respectively examined by DLS (Fig. 2c). The data showed that the average size of 6MP-AuNPs-RDP was slightly larger than that of 6MP-AuNPs (24.6 vs 20.5 nm), while zeta potential of the former was significantly higher than the latter (− 25.8 vs − 37.2 mV), suggesting that the cationic RDP increased the surface potential of the particle. The TEM images showed that both of these nanoparticles were spherically shaped (Fig. 2d).

### Cell Uptake of the Nanoparticles

**Fig. 3:**
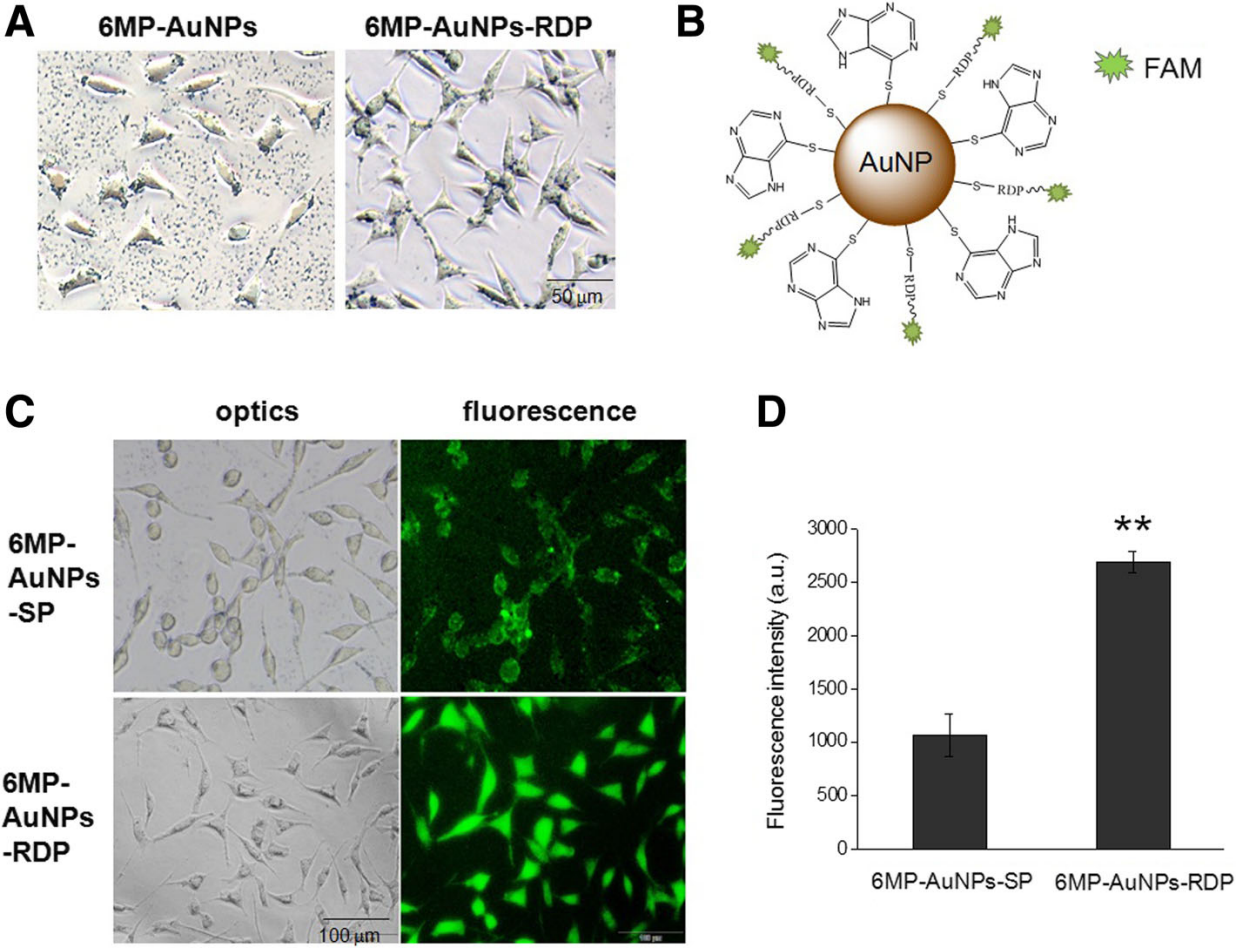
Cell uptake of the nanoparticles. **a** 6MP-AuNPs and 6MP-AuNPs-RDP solutions of 0.25 μg/mL were respectively adjusted to pH 7.4 and added into the cell media for a 30-min incubation. The particles were internalized into SH-SY5Y cells or precipitated on well bottoms. **b** Schematic diagram of 6MP- and fluorescence-labeled peptide-modified AuNP. After the SH-SY5Y cells were incubated with the particles for 2 h, images were taken (**c**) and fluorescence intensity (**d**) was measured. Data are presented as mean ± SEM. The values were averaged for four independent experiments. ^**^p < 0.01 compared with the 6MP-AuNPs-SP-treated cells

When the particle solutions were adjusted to pH 7.4 and added into the cell media, the particles began aggregation and gradually sank to the bottom of wells. However, 30 min later, an obvious blank plaque appeared around the cells, and the gap of 6MP-AuNPs-RDP-treated cells had fewer particle aggregation than that of 6MP-AuNPs (Fig. 3a). Also, more nanoparticles were observed inside the cells treated with 6MP-AuNPs-RDP compared to the cells treated with 6MP-AuNPs.

To further identify that the particles could enter the neuronal cells, fluorescence-labeled peptides were conjugated with the particles (Fig. 3b). After 2 h of incubating the cells with these nanoparticles, a strong fluorescence was observed in the 6MP-AuNPs-RDP-treated cells, while a relatively weak green fluorescence was visible in the 6MP-AuNPs-SP-treated cells. (Fig. 3c). The result of fluorescence intensity measurement with Fluorescence Spectrometer (Hitachi Ltd. Co. Tokyo, Japan) showed that 6MP-AuNPs-RDP-treated cells had significantly higher fluorescence intensity than that of the 6MP-AuNPs-SP-treated cells (Fig. 3d), suggesting that RDP, a type of cell-penetrating peptides (CPPs), could increase the cellular uptake efficiency of the particle.

### Effects of the Nanoparticles on Neuronal Growth

**Fig. 4:**
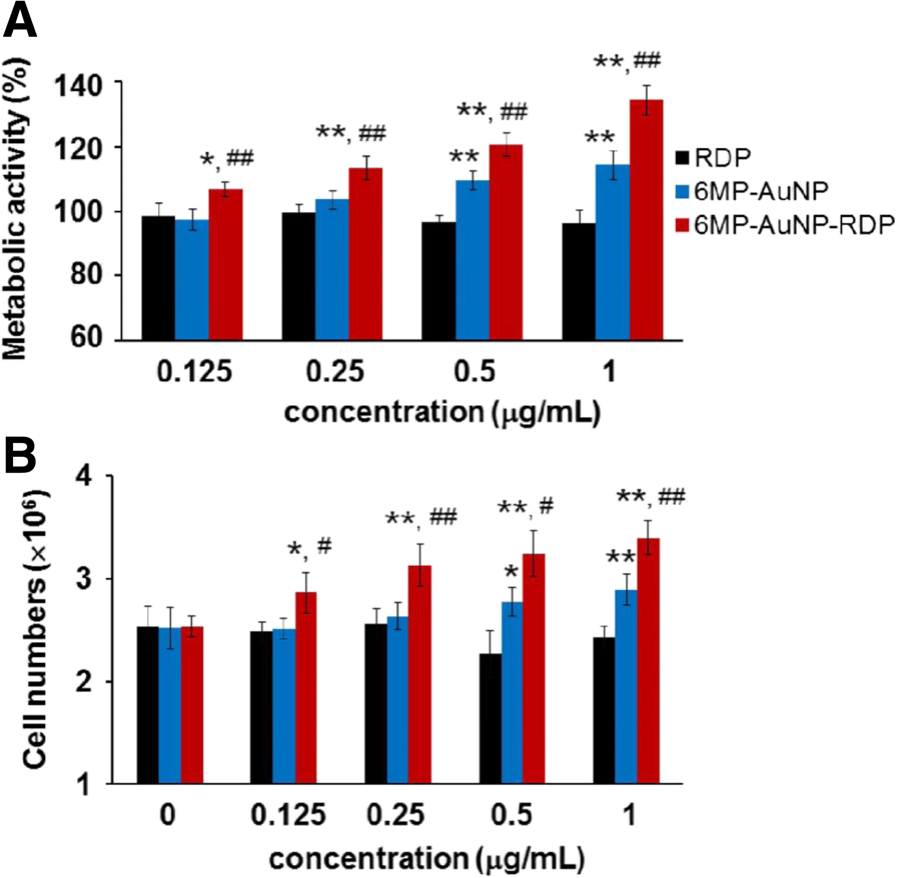
The particles increased cell metabolic activity (**a**) and numbers (**b**), and concentration of 6MP-AuNPs and 6MP-AuNPs-RDP varied from 0 to 1.0 μg/mL Data are presented as mean ± SEM. The cells with only solvent {0.1 M NaOH (pH 9.0) is adjusted to pH 7.4 by 0.1 M HCl} were used as control. The values were averaged for four independent experiments. ^*^p < 0.05, ^**^p < 0.01 compared with RDP-treated cells, ^#^*p* < 0.05, and ^##^*p* < 0.01 compared with the 6MP-AuNPs-treated cells

To examine whether the particles had effects on neuronal growth, both MTT assay and cell counting were used to measure cell metabolic activity and numbers after incubation cells with the particles for 24 h. The results indicated that RDP alone did not affect cell growth, while 6MP-AuNPs-RDP and 6MP-AuNPs at the respective concentration above 0.125 and 0.5 μg/mL, increased cell metabolic activity and cell numbers in a dose-dependent manner (Fig. 4a, b). Also, 6MP-AuNPs-RDP-treated cells showed higher metabolic activity than 6MP-AuNPs-treated cells, which was probably related to the higher cell-penetrating efficiency of 6MP-AuNPs-RDP than 6MP-AuNPs.

### Effects of the Nanoparticles on Neurite Length

**Fig. 5:**
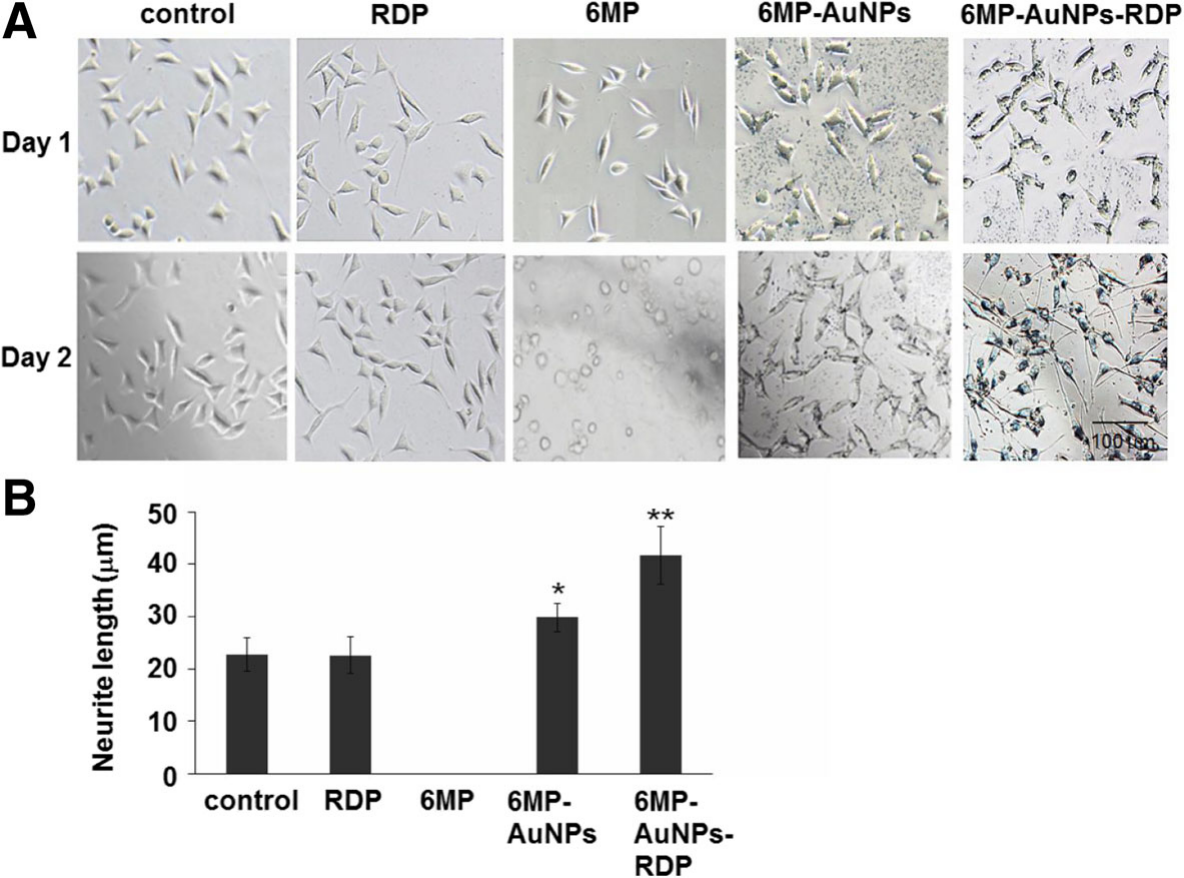
The nanoparticles promoted neurite growth. Images (**a**) and neurite length (**b**) after the cells were respectively treated with RDP, 6MP-AuNPs, and 6MP-AuNPs-RDP for 24 h. The cells with only solvent were used as control. The values were averaged for 200 neurites. ^*^p < 0.05 and ^**^p < 0.01 compared with the control

Besides that the particles could increase cell metabolic activity, impact of the particles on neurite length was also observed at high concentration (1 μg/mL) of the particles. The images (Fig. 5a) showed that the aggregated nanoparticles settled at the bottom of the wells, and a larger blank plaque area appeared around the 6MP-AuNPs-RDP-treated cells.

After the 24-h incubation, the results showed that the cells treated with the particles had longer neurite than the control cells, while there was no significant difference between RDP-treated cells and the control. In addition, the neuritis of 6MP-AuNPs-RDP-treated cells was remarkably longer than that of 6MP-AuNPs-treated cells (Fig. 5b).

From the result of Fig. 5, 6MP killed all the SH-SY5Y cells due to its cytotoxicity; thus, we did not use 6MP to coat plate. Also, as displayed by Figs. 3, 4, and 5, relative small amount of 6MP-AuNPs entered cells, and both neurite length and cell numbers were obviously lower than 6MP-AuNPs-RDP. Therefore, 6MP-AuNPs-RDP was chosen for the further study to examine the effect on cell growth.

### Cell Proliferation and Neurite Growth After Repeated Administration of 6MP-AuNPs-RDP

**Fig. 6:**
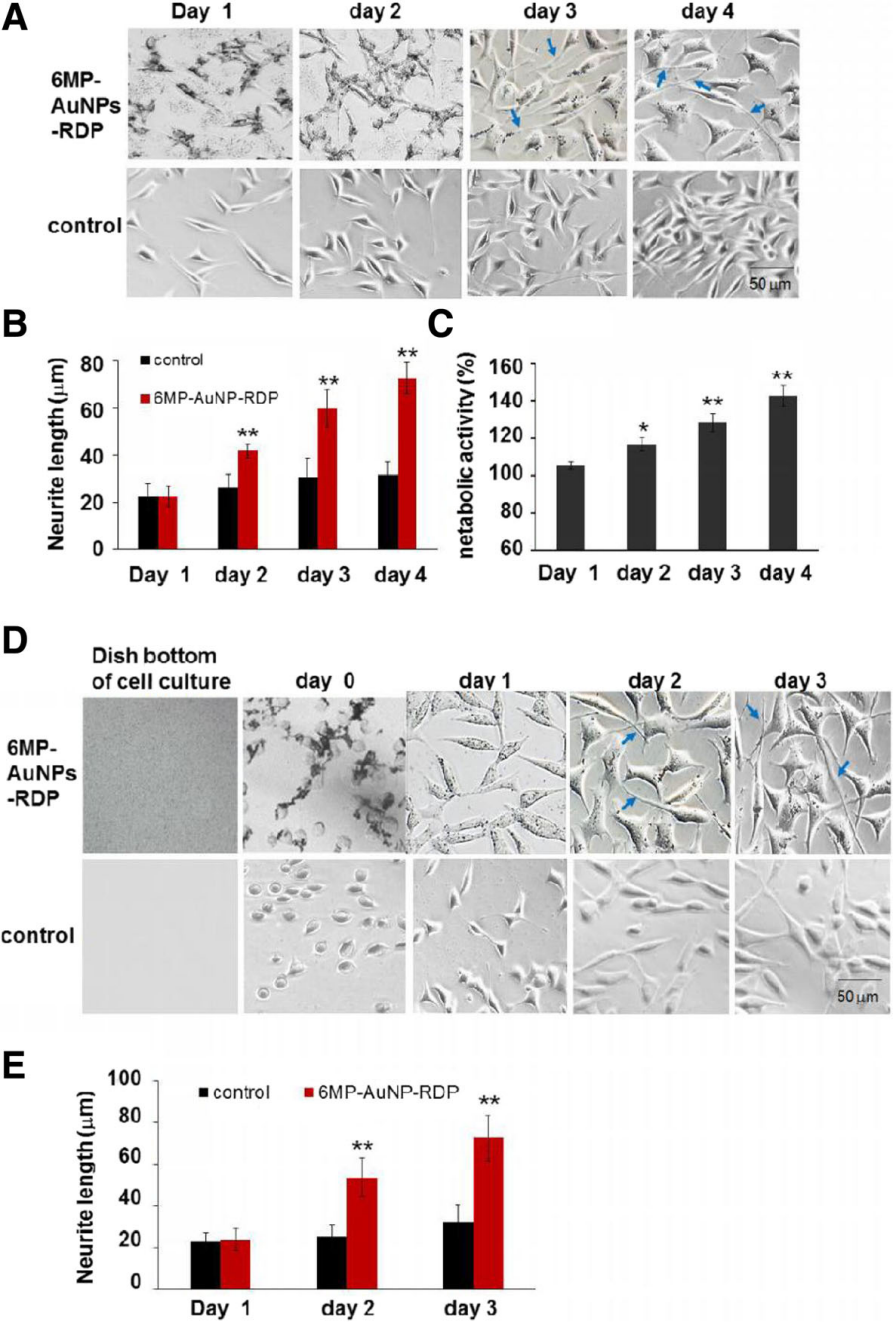
The nanoparticle induced cell proliferation and neurite growth. **a** cell images at days 1, 2, 3, and 4. **b** Neurite lengths of 6MP-AuNPs-RDP-treated cells and the control. The values were averaged for 200 neurites. ^**^p < 0.01 compared with the control. **c** Metabolic activity of 6MP-AuNPs-RDP-treated cells. The cells received treatment three times once a day for 3 days. At the first time, 6MP-AuNPs-RDP was added into the cell media after the cells were cultured for 24 h from cell transplantation. Each concentration of the particles was 0.25 μg/mL. **d** Images of neurite growth of the cells on surface coating with 6MP-AuNPs-RDP. 6MP-AuNPs-RDP was plated on the bottom of culture dishes of 3.5 cm diameter, and then, the cells were transplanted on the dishes. **e** The neurite length were measured at 0 ~ 3 days. The cells with only solvent were used as control. The values were averaged for 200 neurites. ^**^p < 0.01 compared with the control, ^*^p < 0.05, ^**^p < 0.01 compared with the cells of day 1. The blue arrow points to the representative neurites

To further identify the results of 6MP-AuNPs-RDP on cell proliferation and neurite growth, 6MP-AuNPs-RDP were added into the cell media for three times on days 1, 2, and 3 after culturing cells in 6-well plates (day 0). The results showed that neurite length of the particle-treated cells became obviously longer than that of the control (Fig. 6a, b), and the cell metabolic activity increased when the cells were treated with the particles (Fig. 6c).

To examine the effect of 6MP-AuNPs-RDP on neurite growth when the particle was used as a surface coating material, the bottom of culture dishes was coated with 6MP-AuNPs-RDP, and then, the cells were transplated on the coatings. The images showed that the particles quickly attached to the cell membrane (Fig. 6d), then the particles were internalized and distributed in the whole cells, including cytoplasm, nucleus membrane, and cell nucleus. The results also showed that the particle-treated cells had significantly longer neurites than the control (Fig. 6e).

### Effects of the Nanoparticle on Cell Growth After Frozen Storage

**Fig. 7:**
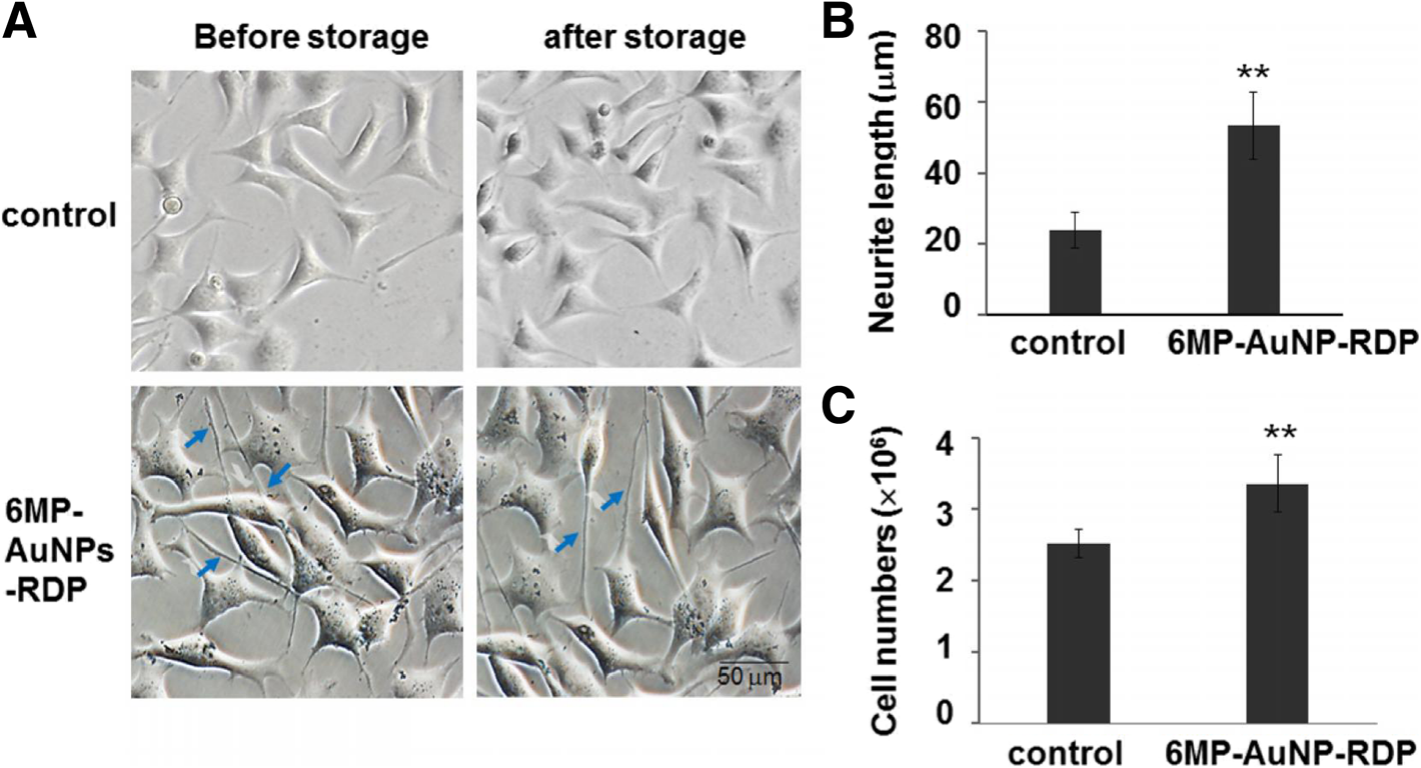
The nanoparticle increased neurite length and cell numbers after frozen storage. (**a**) The cells were treated with 6MP-AuNPs-RDP (1.0 μg/mL) before storage, and the cells were recovered and continued to culture for 3 days. Neurite length (**b**) and cell numbers (**c**) of 6MP-AuNPs-RDP-treated cells were significantly increased after storage. The cells with only solvent were used as control. Data are presented as mean ± SEM. ^**^p < 0.01 compared with the control. The blue arrow points to the representative neurites

To examine the cell tolerance for the particles and identify whether the cells maintained proliferative capacity after the detached growth state, 6MP-AuNPs-RDP-treated cells were frozen and stored for several days when they reached the exponential growth phase. Then, the cells were recovered and cultured on 24-well plates (Fig. 7a). The results showed that the numbers of the particle-treated cells significantly increased and the neurites became longer than the control (Fig. 7b, c), suggesting that the growth of the particle-treated cells could not be affected by a frozen and recovery process.

## Discussion

AuNPs have shown great potential applications in various fields of chemistry, physics, materials, biology, medicine, and related interdisciplinary areas. In order to stabilize the structure of AuNPs, most frequently thiol-modified ligands were used as stabilizing agents which could bind to the surface of AuNPs by the formation of Au-S bonds. In the study, thiol groups of 6MP and RDP were conjugated with the surface of AuNPs, and the particle structure was stable and could be used for further study.

As can be seen from the results, the particles possessed an obvious acid-base property, which was related to the dissociation of the N(9)-H group of 6MP molecule that took place in a pH range of 10.4 ~ 11.2 in solution, and aggregation occurred when the pH value of 6MP-AuNP solution was below 6. The protonation of N9 of the 6MP molecule neutralized 6MP-AuNPs with pH value lower than 6, and then, intermolecular interactions (base stacking interactions) got very strong and compensated the electrostatic repulsion. After RDP modification, the particles showed more complex acid-base behavior because pI of thiol-RDP was about 11.5 besides the acid-base property of 6MP-AuNPs. From titration, the pI value of 6MP-AuNPs-RDP was 7.8, close to the physiological condition (pH 7.4). Thus, 6MP-AuNPs-RDP and 6MP-AuNPs could precipitate from cell media.

In this study, we identified that RDP improved the cell uptake of 6MP-AuNPs, which was consistent with the previous studies. It is commonly known that RDP is a long peptide consisting of 39 amino acids, which is derived from rabies virus glycoprotein that has the capability of transport foreign macromolecules into neuronal cells [15, 16]. In our previous study, cell uptake efficiency of gold nanoclusters was significantly enhanced when the nanoclusters were conjugated with RDP [17]. The mechanism of RDP penetration into cells may be associated with neural cell membrane γ-aminobutyric acid (GABA) receptor- or nicotinic acetylcholine receptor-mediated endocytosis [18, 19].

The study also suggested an opposite effect of 6MP-AuNPs-RDP relative to 6MP that 6MP-AuNPs-RDP promoted cell proliferation and neurite growth, showing obvious neurophic activity. The mechanism of this distinctive difference of 6MP-AuNPs-RDP and 6MP might be associated with the chemical structure of 6MP (a purine derivative of C6 thiol group) [20]. On the surface of 6MP-AuNPs-RDP, thiol group of 6MP was engaged in bonding with AuNPs and thus blocked. Hence, the purine group was exposed on the particle surface. It is well accepted that purine plays a vital role in promoting neuronal growth (such as cell differentiation, neurite formation and extension, synaptogenesis) through intracellular purinergic signaling pathways [21, 22], including mitogen-activated protein kinase/extracellular signal-regulated protein kinase (MAPK/ERK) and phosphatidylinositol 3-kinase/serine-threonine kinase Akt (PI3K/Akt) pathways (the same pathways inducible by neurotrophins and cytokines) [23]. Therefore, the purine of 6MP-AuNPs-RDP might be contributing to the effects of cell proliferation and neurite growth.

It should be pointed out that SH-SY5Y cells showed good excellent tolerance to the 6MP-AuNPs-RDP. When the particle-treated cells received repeated administration of the particles or recovery from frozen storage, even when the cells grew on the surface coated with the particles, they still maintain proliferative activity, suggesting that the 6MP-AuNPs-RDP should have the potential for application.

## Conclusions

Here, we suggested that AuNPs modified with 6MP and RDP could effectively promote cell proliferation and neurite growth. Due to excellent biocompatibility and biosafety of the nanoparticles, they are a promising biomaterial that can be used as a neurophic nanomaterial for neuronal growth.

## Abbreviations

6MP: 6-Mercaptopurine
AD: Alzheimer’s disease
AuNPs: Gold nanoparticles
DLS: Dynamic light scattering
DMEM: Dulbecco’s modified Eagle’s medium
DMSO: Dimethyl sulfoxide
FCS: Fetal calf serum
MTT: 3-(4,5-Dimethylthiazol-2-yl)-2,5-diphenyltetrazolium bromide
PD: Parkinson’s disease
RDP: Rabies virus-derived peptide
TEM: Transmission electron microscopy
